# Anomaly detection in IoT-based healthcare: machine learning for enhanced security

**DOI:** 10.1038/s41598-024-56126-x

**Published:** 2024-03-11

**Authors:** Maryam Mahsal Khan, Mohammed Alkhathami

**Affiliations:** 1 Department of Computer Science, CECOS University of IT and Emerging Sciences, Peshawar, 25000 Pakistan; 2 Information Systems Department, College of Computer and Information Sciences, Imam Mohammad Ibn Saud Islamic University (IMSIU), Riyadh, 11432 Saudi Arabia

**Keywords:** Anomaly detection, IoT, Security, Machine learning, Deep learning, Pearson correlation coefficient, SMOTE, Imbalanced dataset, Information technology, Computer science

## Abstract

Internet of Things (IoT) integration in healthcare improves patient care while also making healthcare delivery systems more effective and economical. To fully realize the advantages of IoT in healthcare, it is imperative to overcome issues with data security, interoperability, and ethical considerations. IoT sensors periodically measure the health-related data of the patients and share it with a server for further evaluation. At the server, different machine learning algorithms are applied which help in early diagnosis of diseases and issue alerts in case vital signs are out of the normal range. Different cyber attacks can be launched on IoT devices which can result in compromised security and privacy of applications such as health care. In this paper, we utilize the publicly available Canadian Institute for Cybersecurity (CIC) IoT dataset to model machine learning techniques for efficient detection of anomalous network traffic. The dataset consists of 33 types of IoT attacks which are divided into 7 main categories. In the current study, the dataset is pre-processed, and a balanced representation of classes is used in generating a non-biased supervised (Random Forest, Adaptive Boosting, Logistic Regression, Perceptron, Deep Neural Network) machine learning models. These models are analyzed further by eliminating highly correlated features, reducing dimensionality, minimizing overfitting, and speeding up training times. Random Forest was found to perform optimally across binary and multiclass classification of IoT Attacks with an approximate accuracy of 99.55% under both reduced and all feature space. This improvement was complimented by a reduction in computational response time which is essential for real-time attack detection and response.

## Introduction

The Internet of Things (IoT) is a major technology that is the basis of several upcoming applications in the areas of health care, smart manufacturing, and transportation systems. IoT relies on the use of various sensors to gather information about humans, devices, and the surrounding environment. This information is passed to the cloud server regularly and as a result, application administrators can make various decisions to improve the efficiency of applications. Similarly, AI techniques can be utilized to automatically control the applications based on the collected data^1^.

Healthcare is one major application of IoT where patients are provided with wearable devices to collect data related to body vitals. Examples of such data could be body measurements such as oxygen level, blood pressure, sugar level, heart rate, etc. Without using IoT, these vital measurements can not be recorded continuously and sent to the cloud for processing. Thus, IoT-enabled health care is an important use case with a huge impact on human lives.

Since IoT-enabled health care involves the recording and sharing of critical data that is linked to human safety, it is vital to design efficient techniques to make sure that the data recording and sharing are reliable and secure. Healthcare systems can be subject to several security attacks that can lead to a loss of confidence in received data. In several cases, wrong decisions can be made on the malicious data, thus leading to the collapse of IoT-enabled healthcare applications.

There are several types of security attacks in healthcare systems such as Denial of Service (DoS) attack in which malicious users aims to deny the wearable or to share data with the cloud. This can be achieved by sharing incorrect data with high frequency towards the wearable or, thus blocking its access to the wireless medium. Similarly, spoofing is another common cyber attack in which malicious users hide their identity to get access to the critical health-related data of patients. Another example of a cyber attack is a brute force attack that tries to crack the password of users’ wearable devices and gain access to the sensor’s data. In addition, there are many other attacks such as data integrity and eavesdropping that can reduce the reliability of IoT health care applications.

This paper focuses on developing anomaly detection techniques for IoT attacks using the publicly available dataset. Following are the major contributions of the paper.

- The authors in^2^ have applied Machine Learning (ML) algorithms in an imbalanced dataset, producing models with high accuracy and low precision scores. The research motivation is to balance the dataset and train ML algorithms accordingly.
- To evaluate supervised machine learning algorithms across inary (2-Class) and multiclass (8 and 34-Class) representations on the balanced dataset.
- To evaluate the computational response time of machine learning models via feature reduction.
- To determine which features are essential for the generalization of machine learning models.

The paper is organized as follows. "Literature review" Section describes the literature review and recent work done in the area of IoT security and anomaly detection and briefly describes the ML algorithms used in the study and how they are evaluated. The problem of an imbalanced dataset and the strategy to resolve it through oversampling techniques is also included in this section. "Methodology" section describes the system model and utilized IoT attack dataset including the methodology and anomaly detection framework of the current study. The result and discussion are presented in "Results and discussion" section. Finally, conclusions are described in "Conclusion" section.

## Literature review

In this section, we present an overview of different intrusion and cyber-attack detection techniques in an IoT network and provide a brief description of different datasets that are used to analyze these attacks. The section also provides information on the Machine learning (ML) algorithm used in the study along with the standard performance metrics used for the evaluation of the ML models. Finally, the section describes the problem with ML models trained on imbalanced datasets and strategies to overcome them.

### Review of different intrusion detection techniques

Table 1 lists different intrusion detection techniques focused on IoT networks. In^3^, authors utilize Deep Neural Network (DNN) and Bi-directional Long Short-Term Memory (Bi-LSTM) techniques to identify the abnormalities in the data. A key feature of the proposed technique is the use of the Incremental Principal Component Analysis (IPCA) technique for reducing the features in the dataset. The proposed technique also uses dynamic quantization for efficient data analysis. The work achieves improved accuracy of intrusion detection and reduced complexity of the model.

**Table 1:** Recent work related to Cyber attack and intrusion detection..

References	Goal	Key idea	Results
^3^	Intrusion detection	DNN	
		Bi-LSTM	Improved accuracy of detection
		IPCA for feature reduction	Reduced complexity of model
		Dynamic quantization	
^4^	Attack detection	Federated learning	
		DNN	Improved accuracy
		Feature reduction	Improved privacy
		Data balancing	
^5^	Intrusion detection	Feature reduction	Improved F1 score
		Data balancing	
^6^	Attack detection	Class imbalance problem	Improved accuracy
		Bagging classifier	Improved precision
		DNN with balanced data	
^7^	Intrusion detection	Adaptive recommendation system	Improved intrusion detection
		Self improving mechanism	
		Autonomous intrusion knowledge	
		pseudo label based voting	
^8^	Intrusion detection	Explainable AI based DNN	Improved efficiency
		RuleFit	
		Shapley additive explanation	

The work in^4^ is focused on efficient cyber attack detection. The main idea of the proposal is to use federated learning for improved privacy and distributed model development. The proposed technique uses a Deep Neural Network (DDN) for attack detection. The work also contributed towards reducing the features and balancing of the data. Results show that the proposed technique improves the accuracy of attack detection as well as the privacy of the system.

In^5^, another intrusion detection for IoT networks is proposed. The focus of the work is on two key factors, one is removing the redundancy in dataset features, and the second is mitigating the imbalance in the dataset. By using these two factors, the proposed technique improves the F1 score of intrusion detection.

The work in^6^ proposes a cyber-attack detection mechanism. The class imbalance problem is handled by the proposed technique. Authors apply DNN on the balanced dataset to perform training and testing. A bagging classifier mechanism is used to improve the performance of the system. The proposed technique achieves improved accuracy and precision.

In^7^ develops an adaptive recommendation system to improve the efficiency of intrusion detection. The main feature of the proposed technique is the development of a self-improving mechanism that autonomously learns the intrusion knowledge. A pseudo-label-based voting system is also used in the proposed technique, thus resulting in improved intrusion detection performance.

The work in^8^ develops an explainable AI-based intrusion detection system. Authors utilize the DNN technique in conjunction with explainable AI mechanisms such as RuleFit and Shapley Additive Explanation. Results show that the developed model is simple and easier to understand while providing improved efficiency.

### Cyber attack and intrusion detection data sets in IoT

There are various publicly available data sets related to cyber attacks and intrusion detection in IoT as shown in Table 2. In^9^, the CIC IDS 2017 attack data set is provided by the Canadian Institute of Cyber Security. A 5-day network traffic data was collected using CIC Flow meter software. The data included normal traffic as well as different types of attacks such as Denial of Service (DoS), Distributed Denial of Service (DDoS), Brute Force, Cross-Site Scripting (XSS), Structured Query Language (SQL) injection, Infiltration, Port Scan, and Botnet.

**Table 2:** Cyber attack and intrusion detection related datasets..

Dataset name	Organization	Collection methods	Attacks
CIC IDS 2017^9^	Canadian Institute of Cyber Security	CIC flowmeter software 5 day data	DoS
			DDoS
			Brute force
			XSS
			SQL injection
			Infiltration
			Port scan
			Botnet
N-BaIoT^10^	University of California, Irvine	Nine Linux based IoT machines 2 IoT Botnets, BASHLITE and Mirai	ACK
			Scan
			SYN
			UDP flooding
CICIoT^2^	Canadian Institute of Cyber Security	105 IoT machines diverse attacks	33 Attacks
			7 Categories
			DDoS
			DoS
			Recon
			Web-based
			Brute force
			Spoofing
			Mirai
NSL-KDD^11^	Tavallaee et al.	Improved version of KDD dataset removed duplicates	DoS
			User to root
			Root to local
			Probing
UNSW_NB-15^12^	University of New SouthWales	Synthetic attack environment normal traffic abnormal synthetic traffic	Fuzzers
			Analysis
			Backdoors
			DoS
			Exploits
			Generic
			Reconnaissance
			Shellcode
			Worms
BoT-IoT^13^	University of New SouthWales	Realistic environment of traffic normal traffic Botnet traffic	DoS
			DDoS
			OS
			Service scan
			Keylogging
			Data exfiltration

The N-BaIoT data set in^10^ was collected by the University of California, Irvine. Nine Linux-based IoT machines were used to generate traffic. Two IoT Botnets were used, one was BASHLITE and the other was Mirai. The generated security attacks included Acknowledgement (ACK), Scan, Synchronize (SYN), and User Datagram Protocol (UDP) flooding.

In^2^, the CICIoT data set was provided by the Canadian Institute of Cyber Security. 105 IoT machines were used to generate diverse security attacks. The generated attacks were divided into 33 attacks and 7 major categories.

The NSL-KDD data set^11^ was provided by Tavallaee et al. The data set is an improved version of the KDD data set and removes duplicate entries. The attacks included in the data set are DoS, User to Root, Root to Local, and Probing.

In^12^, the UNSW_NB-15 data set was provided by the University of New South Wales. A synthetic attack environment was created including normal traffic and synthetic abnormal traffic. Several attacks were generated including Fuzzers, Analysis, Backdoors, etc.

Another data set named BoT-IoT was generated by the University of New South Wales^13^. This data set was based on a realistic environment of traffic containing both normal as well as Botnet traffic. The attack traffic included DoS, DDoS, Operating System (OS), Service scan, keylogging, and data exfiltration.

#### Motivation to use CICIoT 2023 dataset

The author^2^ introduced the CICIoT2023 dataset, which is composed of thirty-three different attacks (categorized into seven classes) executed against 105 IoT devices with well-documented processes defined. So far, the study provides a comprehensive and wide variety of attack types as compared to other reported in literature. Moreover, the main motivation of using the CICIoT2023 dataset is that it has been released recently and there exist only one publication using the dataset. In^3^ only two attacks (Mirai, DDoS) were focused on the study. There exists no article on the use of various intelligent machine learning models in identification of all types of malicious anomalous IoT attacks namely DDoS, DoS, Recon, Web-based, brute force, spoofing, and Mirai. The present study hence contributes to this direction.

### Machine learning algorithms

There exist numerous supervised, unsupervised, and reinforcement-based machine learning algorithms. The research study only investigates the application of supervised ML algorithms in IoT attack detection. The performance of five ML algorithms is tested in the present research work and a brief description of these algorithms is provided herewith.

- *Random forest (RF):* Multiple decision trees are combined in the ensemble learning technique known as RF. For the classification task, the RF’s output is the statistical mode while for the regression task, average of the predictions made by each tree. Applications for RFs are numerous and include image analysis, finance, and healthcare. Their usefulness, usability, and capacity to manage high-dimensional data are well-known attributes.
- *Logistic regression (LR):* It is the type of regression that determines the likelihood that an event will occur and is used for classification. Statistics is used to predict a data value given the previous observations of a data set. The output is discrete. LR operates on a logistic sigmoid function, which accepts any real input and outputs an integer between zero and one.
- *Perceptron (PER):* As a linear classifier, the PER performs best in situations when there is a linear separation of the classes. It uses the perceptron learning rule to update its weights and makes adjustments in response to the misclassifications. Simple and effective, the scikit-learn Perceptron class may not converge on datasets that are not linearly separable. Under such circumstances, more sophisticated algorithms, like support vector machines or neural networks should be used.
- *Deep neural network (DNN):* An artificial neural network with several layers between the input and output layers is called a Deep Neural Network (DNN). Deep learning models are a subclass of neural networks distinguished by their capacity to acquire intricate hierarchical data representations. A deep neural network’s layers are made up of linked nodes or neurons, and these layers are generally divided into three categories: input layer, hidden layer, and output layer. Key characteristics of a DNN include the use of non-linear activation function, deep architectures, and backpropagation algorithm for training weights of the network for locating an optimal solution.
- *Adaptive boosting (AB):* AB creates a powerful classifier by combining several weak classifiers. Training instances are given weights by the algorithm, which then iteratively updates them. A weighted sum of the individual weak classifiers yields the final prediction.

### Machine learning performance metrics

In machine learning classification problems, several performance metrics are commonly used to evaluate the performance of a model. These metrics include accuracy, precision, recall, and F1-score, each of which measures different aspects of classification performance.

- *Accuracy*: Accuracy measures how accurately a classification model is applied overall. It determines the proportion of accurately predicted occurrences to all of the dataset’s instances and is mathematically computed using Eq. (1), where
  - TP (True Positives) is the number of correctly predicted positive instances.
  - TN (True Negatives) is the number of correctly predicted negative instances.
  - FP (False Positives) is the number of instances that were actually negative but were incorrectly predicted as positive.
  - FN (False Negatives) is the number of instances that were positive but were incorrectly predicted as negative.
  1 $$\begin{aligned} \text{Accuracy} = \dfrac{TP+TN}{TP+TN+FP+FN} \end{aligned}$$
- *Precision*: Precision measures the accuracy of positive predictions made by the model. It calculates the ratio of true positives to the total number of positive predictions expressed in Eq. (2).
  2 $$\begin{aligned} \text{Precision} = \dfrac{TP}{TP+FP} \end{aligned}$$
- *Recall*: Recall measures the ability of the model to correctly identify positive instances. It calculates the ratio of true positives to the total number of actual positive instances, expressed in Eq. (3).
  3 $$\begin{aligned} \text{Recall} = \dfrac{TP}{TP+FN} \end{aligned}$$
- *F1-score*: The F1-Score is the harmonic mean of precision and recall. It provides a balance between precision and recall and is particularly useful when dealing with imbalanced datasets, expressed in Eq. (4)
  4 $$\begin{aligned} \text{F1score} = \dfrac{2 \times Precision \times Recall}{Precision + Recall} \end{aligned}$$

### Imbalanced datasets

An imbalanced dataset has a distribution of classes (categories or labels) that is severely skewed, indicating that one class has significantly more samples or instances than the other(s). The occurrence of the dataset is frequently seen in machine learning. In binary classification problems, one class is the majority class and the other is the minority class while in multiclass classification, class imbalance can arise when one or more classes have disproportionately fewer samples than the others. In applications where the minority class is of great importance, such as fraud detection, medical diagnosis, and rare event prediction, addressing class imbalance is essential for reliable predictions. Two major concerns in using ML on an imbalance dataset includes^14,15^:

- *Biased model training*: Machine learning algorithms are often biased in favor of the dominant class when one class outweighs the others significantly. The model may prioritize correctly predicting the majority class while ignoring the minority class because its goal is frequently to minimize the overall error. The model may have trouble making precise predictions for the minority class based on unobserved data because it hasn’t seen enough examples from that group resulting in poor generalization of the problem.
- *Misleading evaluation metrics*: In unbalanced datasets, standard accuracy becomes a misleading statistic. Even if a model that predicts the majority class in every instance can still be highly accurate. The sensitivity (true positive rate) of the model for the minority class is fairly low in unbalanced datasets. This indicates that a large number of false negatives could result from the model missing a significant number of cases from the minority class.

Several tactics and strategies can be used to reduce the problems caused by class imbalance. These include resampling techniques such as oversampling of minority class and under-sampling majority class^16^; synthetic data generation techniques like SMOTE^17^, Adaptive Synthetic Sampling(ADASYN)^1^, cluster-based techniques^18^ to name a few. The authors in^2^ have applied Machine Learning (ML) algorithms in an imbalanced dataset, producing models with high accuracy and low precision scores. The motivation of this research is to balance the dataset and then apply the ML algorithms to generate generalized models with marked improvements in the evaluation metrics.

### Synthetic minority over-sampling technique: balanced dataset generation

Synthetic Minority Over-sampling Technique (SMOTE), is a well-known pre-processing approach in the area of machine learning and data preparation that deals with the issue of class imbalance in classification problems. Class imbalance happens when one class in a binary or multi-class classification problem has significantly fewer samples than the other(s), resulting in an inaccurate model that tends to bias the dominant class. To address this problem, Chawla et al. developed the SMOTE algorithm in 2002^17^. It balances the class distribution by creating artificial examples of the minority class, which improves the learning algorithm’s performance and lowers the likelihood of a biased model. Mathematically expressed as in Eq. (5).

5 $$\begin{aligned} \text{synthetic}\_\text{sample} = x + \lambda \cdot (\text{{neighbor}} - x) \end{aligned}$$

where, *x* is the original minority class instance. *neighbor* is one of the k nearest neighbors of *x* within the minority class. $\lambda$ is a random value between 0 and 1, controlling the amount of interpolation.

The SMOTE method has multiple versions, each with unique adjustments to handle various facets of the class imbalance issue. A few variations of the SMOTE algorithm include e.g. Borderline-Smote which applies SMOTE to instances near the decision boundary^19^; ADASYN that generates samples based on the local density of the minority class^1^; SMOTE-with Edited Nearest Neighbour(ENN) which removes noisy samples using ENN^20,21^; SMOTE-Tomek Links combines SMOTE with Tomek Links undersampling technique to remove noisy samples^22^; SMOTE-Boost that combines SMOTE with AdaBoost ensemble method to oversample minority class in each iteration of AdaBoost^23^ for improving performance. Different versions of the SMOTE algorithm provide different strategies for increasing minority class samples and reducing noisy data. In the current research study, the conventional SMOTE algorithm is used as a starting point to observe the change in performance metrics after applying the SMOTE algorithm to the CICIoT dataset.

## Methodology

### CICIoT2023 dataset

**Figure 1:**
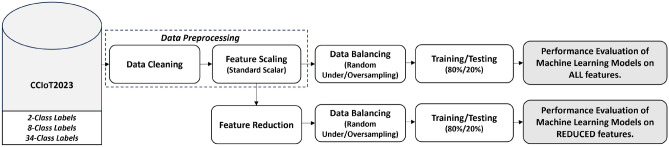
Methodology of the research work applied on the CCIoT2023 Dataset.

In the current research study, we use the publicly available IoT attack dataset namely CICIoT2023^2^. The dataset was created to encourage the creation of security analytics applications for use in actual IoT operations. The authors executed 33 different attacks in an IoT topology of 105 devices. These attacks are classified into seven categories, namely DDoS, DoS, Recon, Web-based, brute force, spoofing, and Mirai. The dataset consists of 169 files in two different file formats PCAP and CSV. The CSV files are PCAP-processed files generating 46 attributes that indicate the different types of attacks. The number of recorded samples per category is not uniform, whereas Web-Based and Brute-Force have far-low representation—a classic sign of an imbalanced dataset. Figure 1 displays the research study’s workflow. The dataset is pre-processed and balanced to ensure credibility in the evaluation of the machine learning models. The data features are further reduced, to improve predictive performance and training times of the ML models across both binary and multiclass representation of the dataset. Further explanation is ahead. The algorithm of the methodology is shown in 1.

**Algorithm 1:**
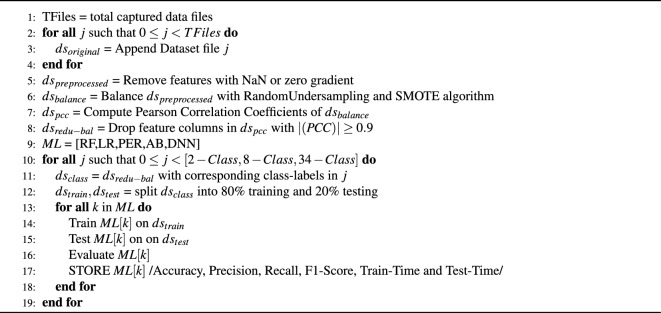
Performance of ML algorithms on balanced representation of CCIoT2023 dataset.

### Dataset preprocessing

Data cleaning is a crucial step in the ML pipeline. Data cleaning includes handling missing or noisy data or dealing with outliers or duplicates. The dataset consists of 33 different classes of IoT attacks with forty-six numerical features. Features with no variation across the thirty-four classes are removed from the dataset. Hence out of 46 features, 40 features are processed ahead. These features are normalized using a standard scalar method which is a common requirement for many machine learning algorithms.

Feature scaling is particularly important for algorithms that use distance-based metrics, as differences in scale can disproportionately impact the influence of certain features on the model. This pre-processing step helps in improving the performance and convergence of ML algorithms. There are two methods of scaling the features in a dataset (1) Normalization (2) Standardization. Normalization is the process of scaling the features within a certain range e.g. [0–1] and standardization is the process of scaling features to a mean of zero and standard deviation of 1. Many of the ML algorithms including linear regression and Neural networks converge faster in the standardized feature space. In the current study, the forty features obtained after cleaning are normalized using a standard scalar method.

### Data balancing

This is the important block of the methodology and requires balancing the dataset using either random undersampling or oversampling via the conventional SMOTE algorithm, described in "Synthetic minority over‑sampling technique: balanced dataset generation" section. The process of dataset generation for binary and multiclass classification is explained below.

- *2-Class representation*: In this scenario, the thirty-three malicious classes are labeled as one category ‘Attack’. Approximately 50% of the data, which captures the different types of malicious representations, from each of the 169 CSV files is randomly extracted and a balanced data set is created. No SMOTE algorithm is used in this particular scenario. The total number of samples per class in the integrated dataset was 8450.
- *8-Class representation*: The data samples from all the different type of attacks i.e. 34 subcategories has been used in the construction of the 8 Class dataset. The process of random undersampling in the majority class and SMOTE-based upsampling of the minority class is executed to produce a uniform representation of the dataset samples. The total number of samples per class in the integrated dataset was 33,800.
- *34-Class representation*: For the 34 classes in the CICIoT dataset, it has been found that two classes namely BruetForce and Web-based have less representative samples in the dataset. The process of random undersampling in the majority class and SMOTE-based upsampling of the minority class is executed to produce a uniform representation of the dataset samples. The total number of samples per class in the integrated dataset was 84,500.

The IoT topology deployed to produce the CICIoT2023 dataset comprises 105 IoT devices. 33 different types of IoT attacks were modeled. In the dataset, the number of rows captured per attack is not uniform, e.g. the attack type DDoS-ICMP Flood contains 7,200,504 data rows representing a majority class whereas WebBased-Uploading Attack is a minority class with 1252 data rows. Applying ML algorithm directly on an imbalanced dataset with non-uniform data-rows across the different attack classes would impact the generalization and performance of a ML model e.g. the authors in^2^ have produced models with high accuracy and low precision scores. Hence, the main motivation and contribution of this research is to balance the dataset and generate ML models that are unbiased with non-misleading evaluation metrics.

### Feature reduction

For feature engineering, model selection, and general data analysis in machine learning, the Pearson correlation coefficient (PCC) is significant since it offers a clear indicator of the relationship between variables. PCC facilitates the creation of more accurate predictive and descriptive models by assisting in the decision-making process over which variables to include in models and how they interact. Many applications have been devised where eliminating highly correlated features has reduced model complexity without compromising the predictive performance. The formula for calculating the Pearson correlation coefficient *r* between two variables, X and Y, with n data points, is given shown in Eq. (6).

6 $$\begin{aligned} r = \frac{\sum _{i=1}^{n} (X_i - \bar{X}) (Y_i - \bar{Y})}{\sqrt{\sum _{i=1}^{n} (X_i - \bar{X})^2 \sum _{i=1}^{n} (Y_i - \bar{Y})^2}} \end{aligned}$$

where $X_i$ and $Y_i$ are the individual data points for variables X and Y respectively and $\bar{X}$ and $\bar{Y}$ are the means of variables X and Y respectively.

**Figure 2:**
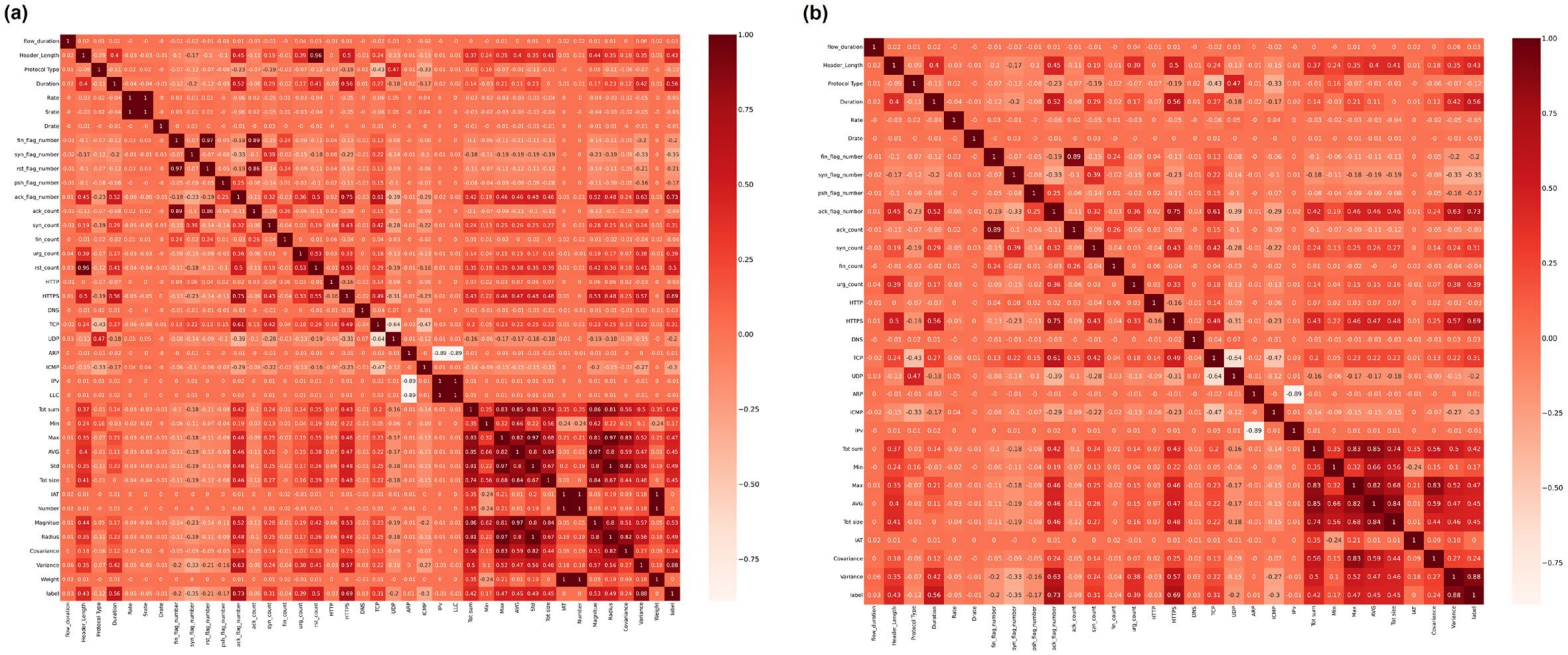
Heat map plot of (**a**) Pearson Correlation Coefficient of forty features in the CCIoT2023 dataset, (**b**) thirty-one Pearson Correlation Coefficients after removing high correlated features, absolute Correlation threshold set to 0.9. Darker shades represent a high absolute correlation coefficient.

As mentioned, the pre-processed dataset consists of forty features. The PCC of the forty characteristics is calculated, and Fig. 2a shows the absolute correlation coefficient heat map. Darker shades in the figure display highly correlated features. A PCC value of 0.9 or higher, in the current study, is regarded as a highly correlated feature, and it is eliminated from the feature collection. Hence a total of thirty-one features are analyzed in the reduced feature space. Referred to Fig. 2b, a heat map of the reduced feature set and related PCC values is displayed.

### Model generation and evaluation

Any binary or multiclass classification problem is modeled through the application of supervised machine learning algorithms. Five popular and powerful supervised ML algorithms (Random forest **RF**, Adaptive Boosting **AB**, Logistic Regression **LR**, Perceptron **PER** and Deep Neural Network **DNN**); are studied on the balanced dataset with both full features and reduced feature set respectively. The datasets are split into 80% training and 20% testing as followed in the research study^2^ for a fair comparison. Standard performance metrics for evaluating supervised algorithms, discussed in "Machine learning performance metrics" section, are computed and reported in Table 3 for 2-Class, 8-Class, and 34-Class respectively.

**Table 3:** Performance of supervised machine learning algorithms on a balanced representation of the CICIoT2023 Dataset with 2-Class, 8-Class, and 34-Class representations, across the full feature set and the reduced feature set..

Classification	Metrics	All features				
		RF	LR	AB	PER	DNN
2 Class	Accuracy	0.99570414	0.98431952	0.99408284	0.97514792	0.98875739
	Precision	0.99500941	0.983730717	0.994089068	0.975531538	0.98878204
	Recall	0.99497041	0.98343195	0.99408284	0.97514792	0.98875739
	F1-score	0.99497031	0.98342939	0.99408282	0.97514291	0.98875725
8 Class	Accuracy	0.95520710	0.63357618	0.48387204	0.51879807	0.80096153
	Precision	0.9553639	0.64638726	0.54242677	0.53682618	0.80409692
	Recall	0.9552071	0.63357618	0.48387204	0.51879808	0.80096154
	F1-score	0.95496382	0.63101848	0.46571153	0.50323578	0.80079878
34 Class	Accuracy	0.96541594	0.55419248	0.45970240	0.42658371	0.72217716
	Precision	0.9649844	0.55479577	0.60071804	0.49572711	0.74045282
	Recall	0.96541594	0.55419248	0.4597024	0.42658371	0.72217717
	F1-score	0.96482905	0.53594043	0.44796463	0.40393062	0.71522424

Classification	Metrics	Reduced features				
		RF	LR	AB	PER	DNN
2 Class	Accuracy	0.99556213	0.98343195	0.99437869	0.9742603	0.98934911
	Precision	0.99559146	0.98362770	0.99439272	0.97426451	0.98941766
	Recall	0.99556213	0.98343195	0.99437870	0.97426036	0.98934911
	F1-score	0.99556206	0.98343028	0.99437866	0.97426030	0.98934874
8 Class	Accuracy	0.95545118	0.67647928	0.45780695	0.5661020	0.83071745
	Precision	0.95559533	0.69044049	0.49852236	0.58018594	0.83629505
	Recall	0.95545118	0.67647929	0.45780695	0.56561021	0.83071746
	F1-score	0.95515475	0.6739131	0.42244699	0.56370658	0.83003669
34 Class	Accuracy	0.96327706	0.56593456	0.44914201	0.47258614	0.81235816
	Precision	0.96281357	0.57223999	0.52861758	0.51726442	0.8224986
	Recall	0.96327706	0.56593456	0.44914201	0.47258615	0.81235816
	F1-score	0.9626063	0.54896777	0.40313942	0.45495343	0.80931119

RF Models performed best under all categories.

## Results and discussion

Table 3, shows the performance of ML algorithms on the balanced dataset across three defined classification scenarios i.e. 2-Class, 8-Class, and 34-Class. The ML models generated are evaluated based on Accuracy, Precision, Recall, and F1-Score details which have been explained in "Machine learning performance metrics" section. Overall, **RF** has been found to perform better than other ML models across the different scenarios. In the 2-Class task, all of the ML models perform with an accuracy of $\ge 98\%$, while it decreases with increasing complexity of the problem i.e. 8-Class and 34-Class label identification. There is a slight improvement in accuracy for the ML models trained in the reduced feature e.g. 0.06% in RF and DNN models. With balanced dataset representation across the three classification tasks, improvement in precision, recall, and f1-score from the ones reported in literature^2^ is obtained.

**Figure 3:**
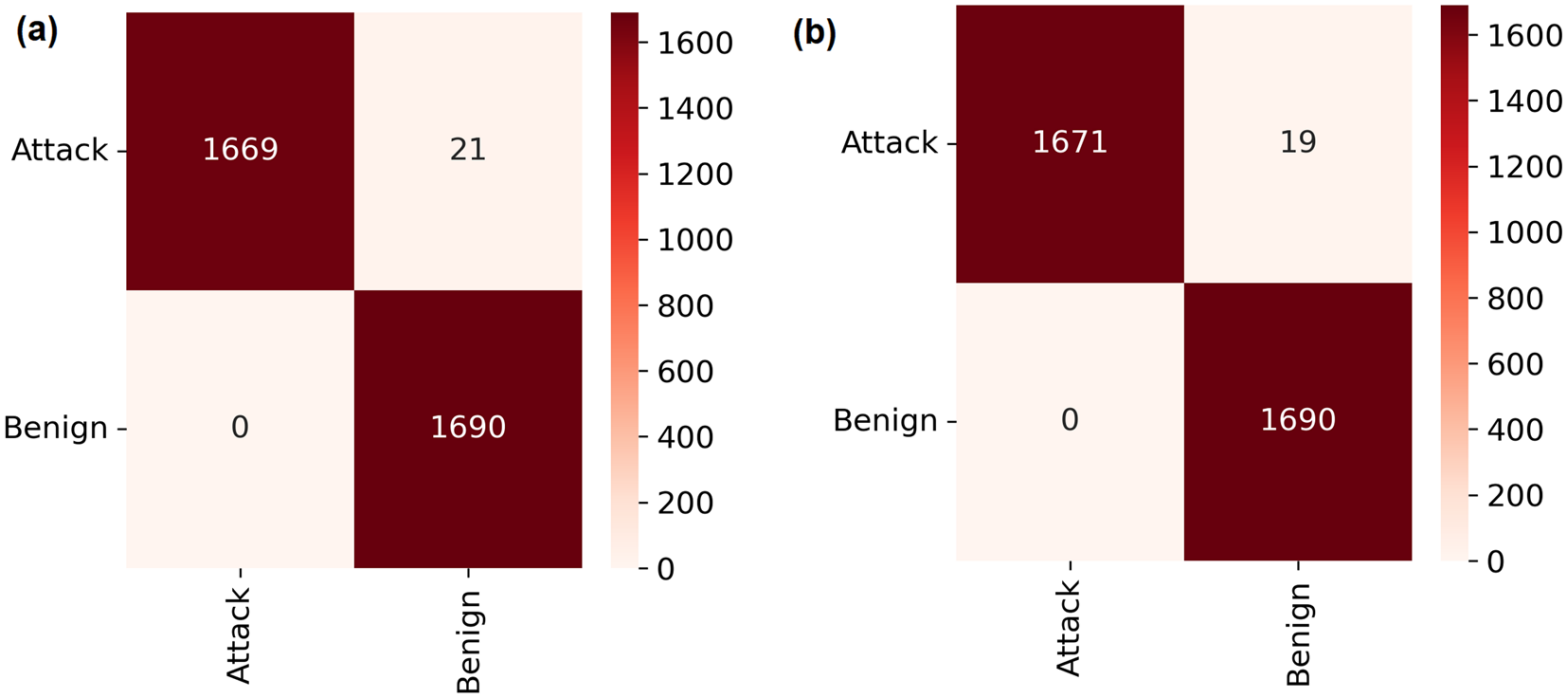
Confusion matrices of RF models on a binary classification task i.e. Attack versus benign, using the CICIoT2023 dataset across (**a**) all features and (**b**) reduced features, respectively.

To visualize the performance of the RF models across the different class categories, confusion matrices are observed. In Figure 3, for the binary classification problem, out of the 1690 test samples per category i.e. benign or attack, benign prediction is found to be more accurate than the attack ones in both scenarios. This might be attributed to the fact that the 33 variations of attack are labeled as one category. The f1-score of the RF-model is found to slightly improve in the reduced feature space i.e. from 99.49 to 99.55% respectively.

**Figure 4:**
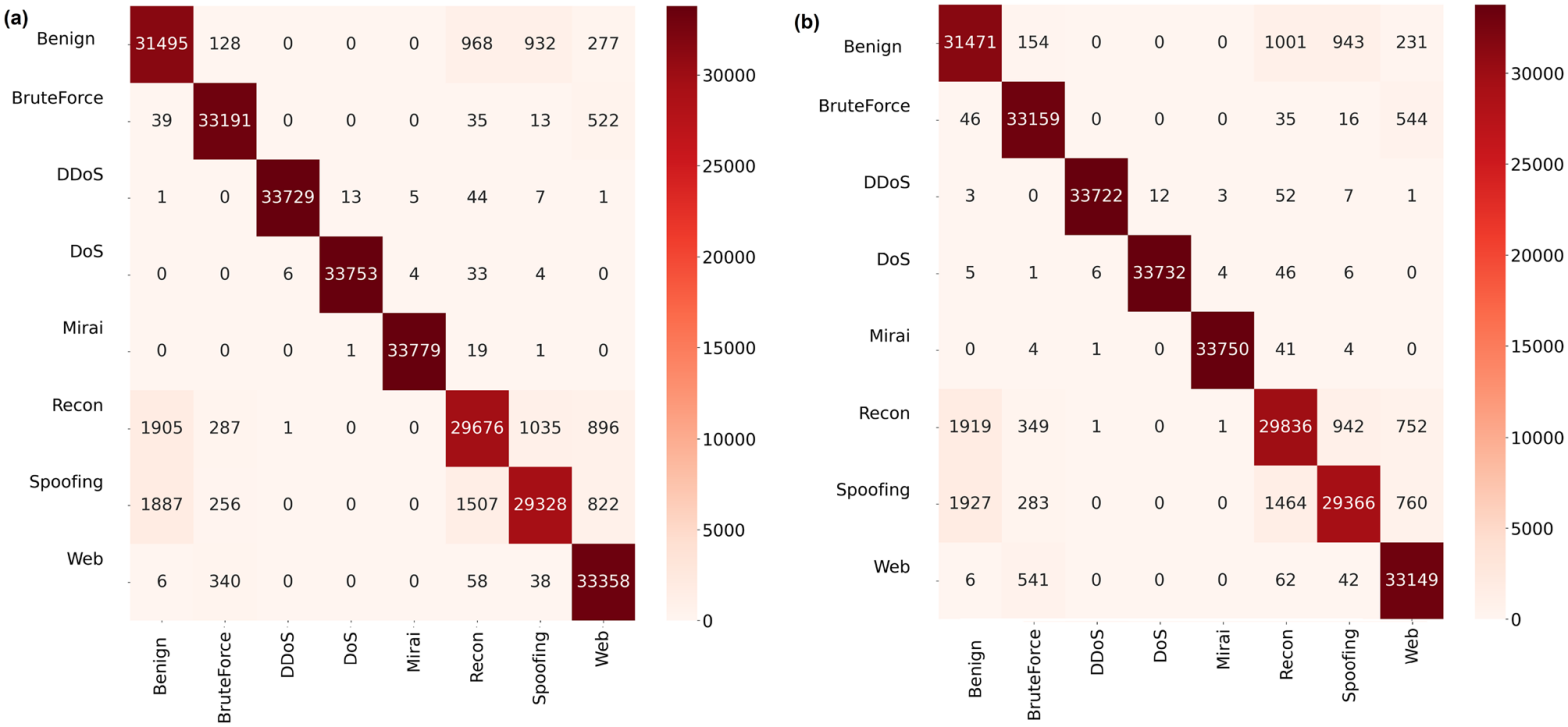
Confusion matrices of trained RF models on a multiclass classification task with 8-class labels, using the CICIoT2023 dataset across (**a**) all features and (**b**) reduced features, respectively.

Figure 4 shows the confusion matrices of the multi-classification eight-class problem where 33,800 samples per category were tested by the RF model under both scenarios. Two attack categories in particular *Recon* and *Spoofing* were found to be poorly recognizable (with an f1-score of 90%) by the RF models despite being trained on real samples. SMOTE-based synthetic samples generated for *BruteForce* and *Web* were found to be in good agreement with the original training samples. Further analysis is required to understand *Spoofing* and *Recon* attack characteristics.

**Figure 5:**
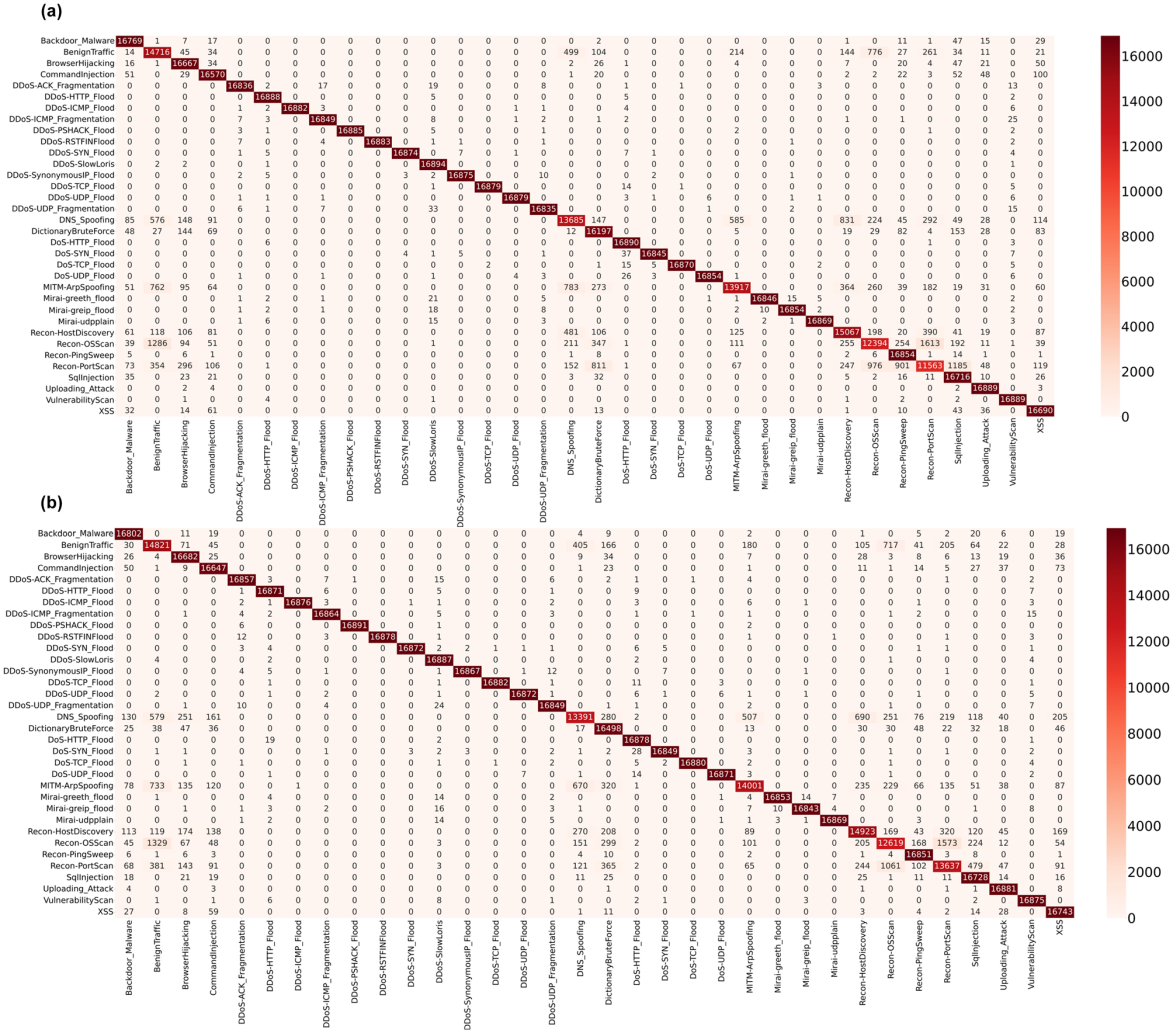
Confusion Matrices of trained RF models on a multiclass classification task with 34-class labels, using the CICIoT2023 dataset across (**a**) all features and (**b**) reduced features, respectively.

In the multi-classification 34-class problem, 16,900 samples per category were tested. Confusion matrices for the RF models under both scenarios (all features and reduced features) are shown in Fig. 5. In the test set, 16,900 samples per category were tested on the trained model. 31 of the classes produced an f1-score greater than 85% while three classes, *DNS-Spoofing*, *Recon-PortScan* and *Recon-OSScan* had an f1-score of 83%, 82% and 79%. These subclasses belong to *Recon* and *Spoofing* IoT attack category, which was also found harder to classify than other class labels in the 8-Class task.

**Figure 6:**
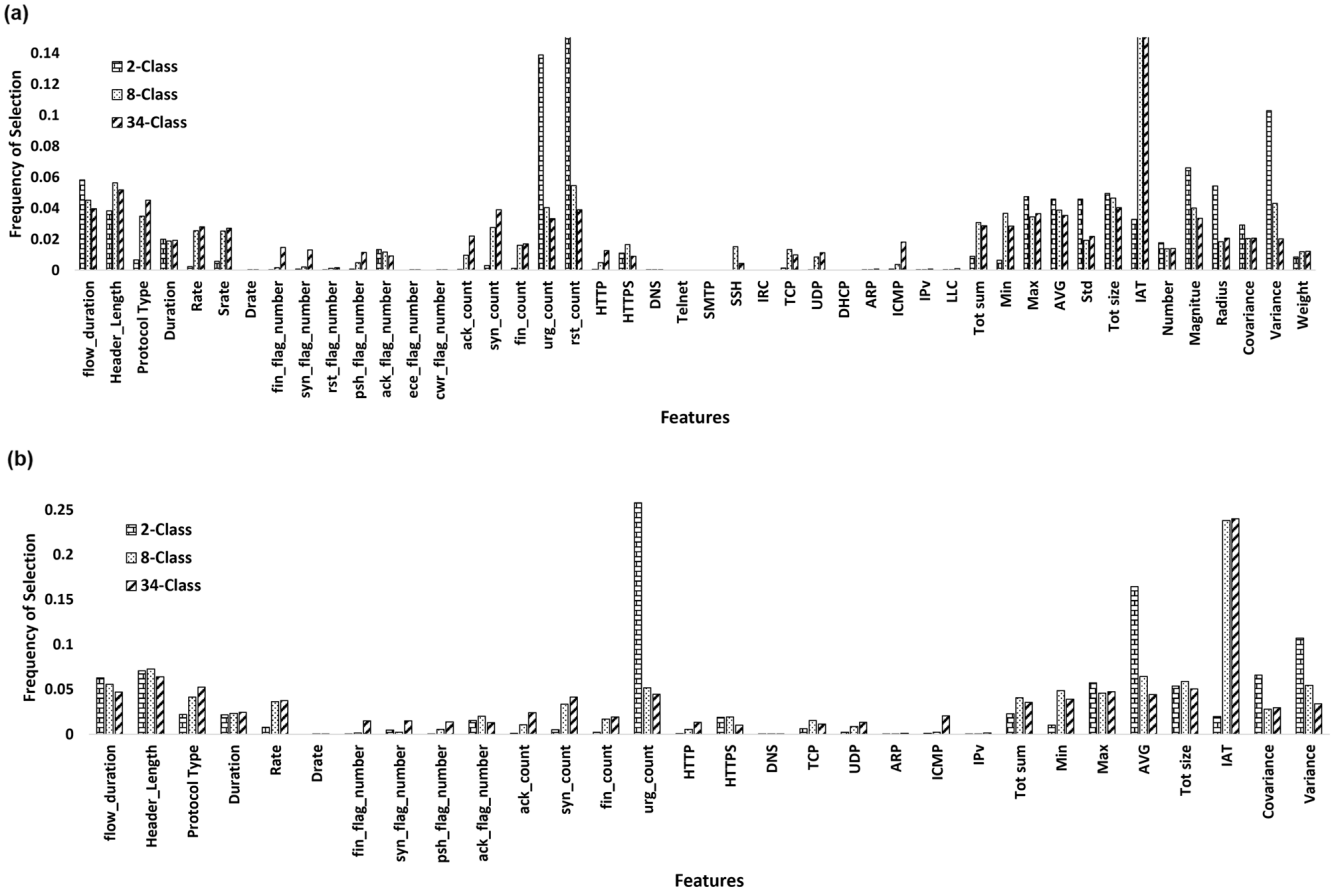
Feature significance graphs, extracted from the RF models across (**a**) all features and (**b**) reduced features in the CCIoT2023 Dataset for 2-Class, 8-Class and 34-Class classification tasks.

An additional tool for comprehending important characteristics in the dataset is a feature importance graph, which is produced through RF models. The feature significance graph from the RF models for the three classification tasks is displayed in Fig. 6, where (a) shows the RF models when all features are used and (b) shows the RF models when a reduced feature set is used. The top features identified in the binary classification tasks under both scenarios were $urg_{count}$ and *AVG*. $urg_{count}$ is the number of packets with urg flag set and *AVG* represents the average packet length. For both of the multi-classification tasks, *IAT* was found to be the top feature. *IAT* measures the time difference between the current and the previous packet. The statistical measurements e.g. Header Length, Min, Max, Average, covering the right side of the feature graph in Fig. 6 were more frequently chosen than the other features.

**Figure 7:**
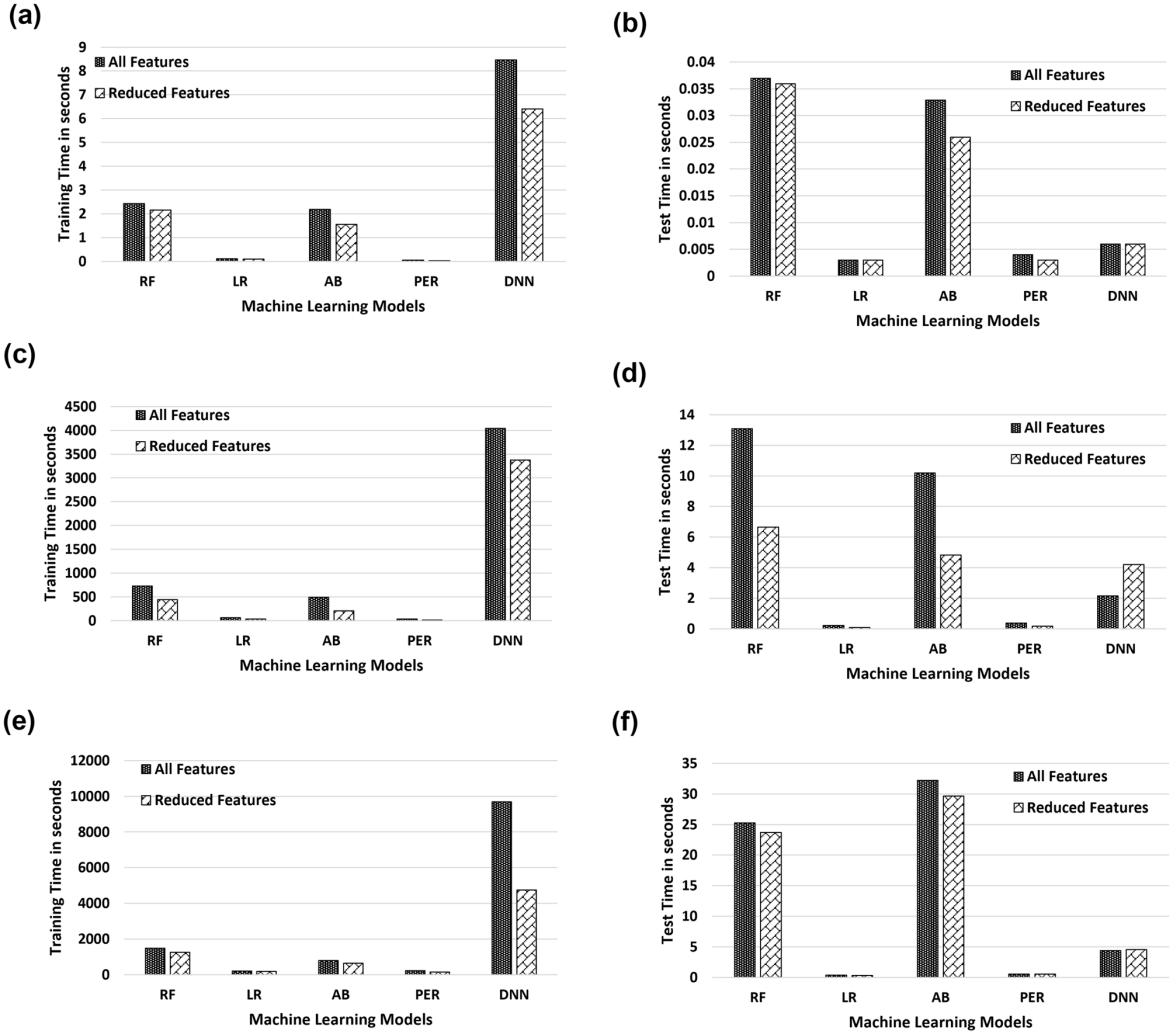
Time is taken, in seconds, to train and test supervised ML algorithms, with and without feature reduction. The figure shows training and testing time for (**a**, **b**) 2-Class, (**c**, **d**) 8-Class, and (**e**, **f**) 34-Class multiclassification tasks, respectively.

Figure 7a, c and e displays the training time in seconds and Fig. 7b, d and f shows the testing time in seconds of the ML algorithms on all and reduced feature sets for 2-Class Fig. 7a and b, 8-Class Fig. 7b and c and 34-Class classification Fig. 7e and f tasks respectively. As the feature set is reduced, we can see a reduction in the training time of all the models. For the DNN model performance in 2-Class classification, Fig. 7a and b, training time across all features was approximately 8.6s while in the reduced space it was 6.6s respectively. Similarly, as the feature set is reduced in almost all cases there is a reduction in response time of the models. For the RF model in 8-Class classification, Fig. 7d, testing time across all features was approximately 13.08 s while in the reduced feature space was 6.64 s secs respectively. All these steps are carried out in the development environment with Intel Core i7 7820HQ-processor, 32 GB DDR4 RAM, and Windows 10 operating system.

The CICIoT2023 dataset has been recently released and there exists not much literature using the dataset. The reported best models in the study are compared with the best models produced by the authors in^2^ and are shown in Table 4. The optimum performing model metrics are highlighted in bold. The results of the existing study have performed better than the ones reported. The dataset originally was imbalanced hence models generated have low recall values. Recall values can be seen improved due to balancing the data samples across the different classification tasks.

**Table 4:** Performance comparison of the best ML models with others reported in the literature.

Method	Labels	Accuracy	Precision	Recall	F1-Score	References
Random forest	2-Class	**0**.**99680798**	0.965395244	0.965163906	0.965279544	^2^
	8-Class	**0**.**994368173**	0.705407564	0.91001105	0.71928904	
	34-Class	**0**.**99164365**	0.704492066	0.831586401	0.714021981	
Random Forest	2-Class	0.99556213	**0**.**99559146**	**0**.**99556213**	**0**.**99556206**	Current Research
	8-Class	0.95545118	**0**.**95559533**	**0**.**95545118**	**0**.**95515475**	
	34-Class	0.96327706	**0**.**96281357**	**0**.**96327706**	**0**.**9626063**	
DL-BiLSTM	8-Class	0.9313	0.9180	0.9313	0.9194	^24^

The best figures are highlighted in bold.

## Conclusion

The use of Medical Internet of Things (IoT) devices in healthcare settings has made automation and monitoring possible e.g. in enhanced patient care and remote patient monitoring. However, it has also introduced a host of security vulnerabilities and risks including identity theft, unauthorized alteration of medical records, and even life-threatening situations. Furthermore, it is becoming more challenging to secure each device entry point in real-time due to the growing usage of networked devices.

Machine learning has the potential to detect and respond to attacks in real-time by identifying anomalies in the data captured by IoT devices. The current study explored the potential of supervised machine learning algorithms in identifying anomalous behavior on a recently published dataset, CCIoT2023. The dataset consists of 33 different categories of IoT attacks represented by 46 features, with a varying number of data samples. The dataset is imbalanced, i.e., it has a non-uniform sample distribution. The study explored improving machine learning models by employing a balanced approach to data distribution using the SMOTE algorithm. Classification models for three strategies of ‘IoT Attack’, two-class, eight-class, and thirty-four class, were investigated. Random Forest was found to excel in all three defined classification problems and performed better than what has been reported so far in the literature. Eliminating strongly correlated features slightly improved the performance of the model but reduced computational response time and enabled real-time detection.

The feature importance graph depicted $urg_{count}$-number of urg flags in the packet and *AVG*-average packet length in 2-Class and *IAT* – time difference between packet arrival time, as an important feature in discriminating various attack categories in multiclassification problem. Moreover, certain IoT attacks e.g. Spoofing and Recon require further analysis and feature expansion to be able to discriminate these classes and their corresponding sub-classes further.

## Data Availability

Details of data is available in the paper.
